# Macrocytosis may be associated with mortality in chronic hemodialysis patients: a prospective study

**DOI:** 10.1186/1471-2369-12-19

**Published:** 2011-05-11

**Authors:** Karthik K Tennankore, Steven D Soroka, Kenneth A West, Bryce A Kiberd

**Affiliations:** 1 Division of Nephrology, Department of Medicine, Dalhousie University, Halifax, Nova Scotia, Canada

## Abstract

**Background:**

Macrocytosis occurs in chronic hemodialysis (CHD) patients; however, its significance is unknown. The purpose of this study was to establish the prevalence and distribution of macrocytosis, to identify its clinical associations and to determine if macrocytosis is associated with mortality in stable, chronic hemodialysis patients.

**Methods:**

We conducted a single-centre prospective cohort study of 150 stable, adult CHD patients followed for nine months. Macrocytosis was defined as a mean corpuscular volume (MCV) > 97 fl. We analyzed MCV as a continuous variable, in tertiles and using a cutoff point of 102 fl.

**Results:**

The mean MCV was 99.1 ± 6.4 fl, (range 66-120 fl). MCV was normally distributed. 92 (61%) of patients had an MCV > 97 fl and 45 (30%) > 102 fl. Patients were not B12 or folate deficient in those with available data and three patients with an MCV > 102 fl had hypothyroidism. In a logistic regression analysis, an MCV > 102 fl was associated with a higher Charlson-Age Comorbidity Index (CACI) and higher ratios of darbepoetin alfa to hemoglobin (Hb), [(weekly darbepoetin alfa dose in micrograms per kg body weight / Hb in g/L)*1000]. There were 23 deaths at nine months in this study. Unadjusted MCV > 102 fl was associated with mortality (HR 3.24, 95% CI 1.42-7.39, P = 0.005). Adjusting for the CACI, an MCV > 102 fl was still associated with mortality (HR 2.47, 95% CI 1.07-5.71, P = 0.035).

**Conclusions:**

Macrocytosis may be associated with mortality in stable, chronic hemodialysis patients. Future studies will need to be conducted to confirm this finding.

## Background

Anemia is a common consequence of end-stage renal disease (ESRD). Several causes have been identified including iron deficiency [1], reduced production of erythropoietin [2], shortened red cell survival [3] and folate deficiency [4]. Among these, endogenous erythropoietin deficiency is the predominant cause [2].

Erythropoiesis stimulating agents (ESAs) have been effective in treating the anemia of ESRD [5,6]. However, despite their use, anemia may still occur and is associated with significant morbidity and mortality in dialysis patients [7,8]. Furthermore, higher ESA doses have also been associated with increased mortality in hemodialysis patients [9-11]. Whether the increase in mortality observed with ESAs is due to achieved hemoglobin, ESA resistance due to inflammation, a direct effect of erythropoietin, concurrent administration of intravenous iron or some other mechanism, is unclear [12].

Morphologically, the anemia of ESRD is typically normocytic and normochromic [13], but up to 30% [14-16] may have macrocytosis. Proposed causes of macrocytosis in dialysis patients include intravenous iron [15,17], megaloblastic anemia due to B12 and folate deficiency [4] or dialysis-induced changes in red cell volume [18,19]. Reticulocytosis is associated with macrocytosis in the general population [20] and initial studies of ESAs suggested stable and predictable increases in reticulocyte levels with maintenance erythropoietin therapy [21]. However, reticulocytes can also be smaller than mature red cells [22], and no studies have specifically addressed the relationship between ESAs and macrocytosis.

While several studies have suggested causes, no studies have examined for clinical associations with macrocytosis in dialysis patients. Furthermore, an association between red cell size and mortality has not been identified in previous study [23].

In our hemodialysis population, we have observed that many patients have an unexplained macrocytosis. The purpose of this study was to establish the prevalence and distribution of macrocytosis, to identify its clinical associations and to determine if macrocytosis is associated with mortality in stable, chronic hemodialysis patients.

## Methods

**Figure 1:**
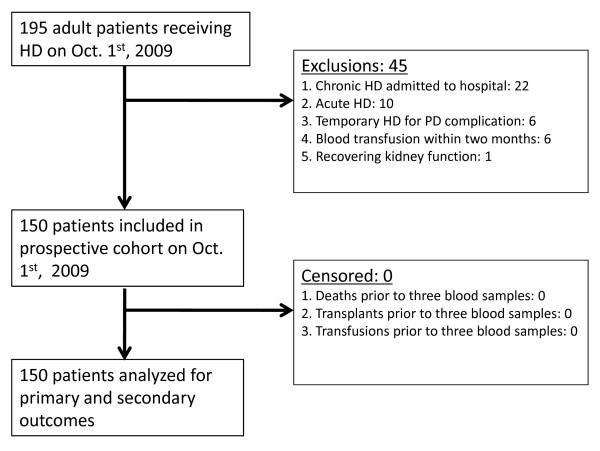
**Prospective cohort selection**.

We conducted a single-centre prospective cohort study of 150 adult patients on chronic hemodialysis (defined as greater than three months). The cohort was derived from all 195 patients undergoing hemodialysis in our local centre on October 1^st^, 2009. Exclusions are noted in figure 1. Three measurements of baseline laboratory data including red blood cell mean corpuscular volume (MCV) were collected monthly from October through December 2009. Values were subsequently averaged to avoid potential laboratory error associated with single measurements. Any individuals that died, were transfused or received a kidney transplant before completing three sets of blood tests were censored prior to study analysis (figure 1). Follow up began from the date of last blood work and patients were prospectively followed for nine months. This study was approved by the Queen Elizabeth II Health Sciences Centre research ethics board, our institutional research ethics committee.

We collected baseline clinical data including age, gender, race, time on dialysis, hemodialysis access, urea reduction rate, cause of ESRD and comorbid medical conditions. Causes of ESRD and comorbid conditions were extracted from existing patient charts and definitions were at the treating physician's discretion. For medical comorbidities, we calculated a Charlson-Age Comorbidity Index (CACI) at baseline in all patients [24]. This index allocates different point scores to 19 individual medical conditions depending on the risk of mortality with each condition. In addition, one point is given for each decade > 40 years of age. Included in this score are common medical comorbidities such as diabetes, congestive heart failure, cancer, dementia and peripheral vascular disease. The CACI has been identified as a mortality predictor in dialysis patients. The lowest mortality is observed at scores ≤ 3 and the highest mortality at scores ≥ 8 [25]. All hemodialysis patients in our unit receive darbepoetin alfa, and either iron sucrose or iron dextran to maintain a target hemoglobin of 110 g/L (range 100-120 g/L). Doses of both monthly intravenous iron and darbepoetin alfa averaged over the first three months were recorded. Medication use including immunosuppressive agents, antiepileptic drugs, insulin, oral folate and multivitamins was also recorded. MCV was measured in femtolitres (fl). MCV was calculated using a Coulter LH 750 hematology analyzer, rounded to the nearest tenth. This analyzer has a within run coefficient of variation of ≤ 0.8 fl [26]. Our laboratory definition of macrocytosis is an MCV > 97 fl (normal range 80-97 fl). Additional baseline laboratory data available included the complete blood count (CBC), electrolytes, calcium, phosphate, parathyroid hormone, ferritin, percent saturation of transferrin (tsat), albumin, alkaline phosphatase, hepatocellular liver enzymes and serum glucose. We also collected reticulocyte counts, serum B12, red blood cell folate and serum thyroid stimulating hormone (TSH) levels available within one year of study onset (October 1^st^, 2009). Finally, we calculated a darbepoetin alfa to hemoglobin (Hb) ratio for each patient, [(weekly darbepoetin alfa dose in micrograms per kg body weight / Hb in g/L)*1000].

The primary outcomes of this study were all cause mortality and time to death in patients with and without macrocytosis at baseline. Causes of death were categorized at the treating physician's discretion according to the Canadian Organ Replacement Registry (CORR) diagnostic codes. CORR uses the ICD-10-CA [27], which is a modified version of the International Statistical Classification of Diseases and Related Health Problems, 10th Revision (ICD-10). In addition to mortality, we also examined for associations of macrocytosis with the clinical and laboratory parameters listed above. Patients were censored at the time of kidney transplantation.

All statistical analyses were performed using SPSS for Windows software (SPSS version 15.0, Chicago, IL). We initially examined MCV both as a continuous variable and using a cutoff point of > 97 fl. Recognizing the potential for a nonlinear relationship between macrocytosis and mortality, we also examined MCV in tertiles using a Kaplan Meier survival analysis. We subsequently used an ROC curve to determine the most statistically significant MCV cutoff point predictive of death. Differences in baseline characteristics using this MCV cutoff point were calculated for continuous and categorical variables. Univariate clinical and laboratory associations were also determined using a binary logistic regression analysis. Spearman correlation coefficients were calculated to determine collinearity between association variables. For highly correlated variables, only those with the highest statistical significance were incorporated into a backward conditional multivariate model. A Cox survival analysis was used to evaluate time to death. Variables associated with macrocytosis and mortality were chosen for incorporation into the multivariate Cox survival analysis.

## Results

### Patient Characteristics

**Figure 2:**
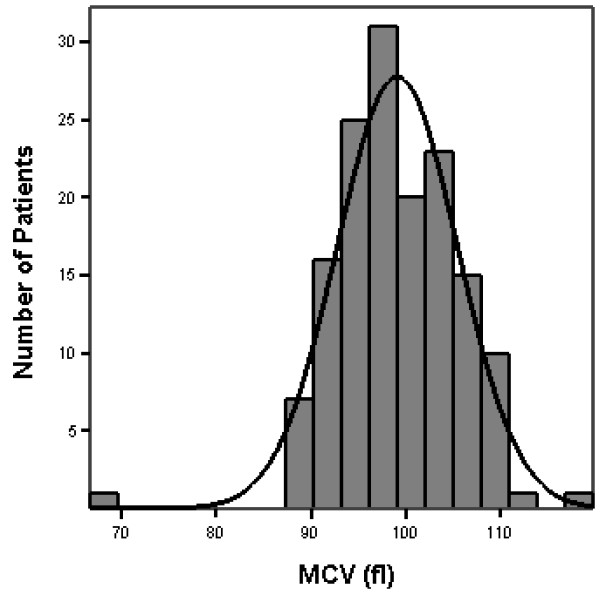
**Distribution of MCV (fl)**. Mean MCV was 99.1 ± 6.4 fl, (range 66-120 fl).

All 150 eligible patients completed three sets of blood tests prior to study analysis (figure 1). The mean MCV (average of three values) for all patients was 99.1 ± 6.4 fl, (range 66-120 fl). MCVs for each of the three measurements were 99.0 ± 6.4 fl, 99.1 ± 6.3 fl and 99.2 ± 6.4 fl with an intra patient variation of 1 ± 0.7 fl. MCV was normally distributed (figure 2). Specifically, 92 (61%) of patients had an MCV > 97 fl, 59 (39%) > 100 fl, 45 (30%) > 102 fl and 24 (16%) > 105 fl. TSH, serum B12 and RBC folate levels were available at baseline in 22 (49%), 21 (47%) and 28 (62%) patients with an MCV > 102 fl. In those patients with available data, serum B12 and RBC folate levels were normal. Three patients had biochemical evidence of hypothyroidism (elevated TSH and low free T4). Reticulocyte counts were only available in a few patients.

### MCV cutoff point most predictive of death

**Figure 3:**
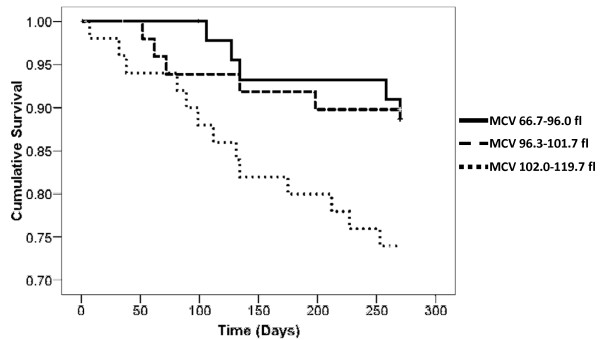
**Kaplan Meier survival in tertiles of MCV (fl)**. Log rank p = 0.047

As a continuous variable in a Cox survival analysis, unadjusted mean MCV was associated with death (HR 1.08 for each 1 fl increase in MCV, 95% CI 1.01-1.15, P = 0.024). An MCV > 97 fl was not predictive of higher mortality, however, a Kaplan Meier survival analysis demonstrated a significant step up in mortality at the highest tertile of MCV (figure 3, log rank P = 0.047). This suggested a nonlinear relationship between MCV and mortality. Using an ROC curve, the optimal MCV cutoff point predictive of death was > 102 fl (area under curve 0.653 ± 0.066, P = 0.019, 95% CI 0.524-0.783). Baseline characteristics using this MCV cutoff point are listed in table 1. Univariate associations with an MCV > 102 fl are listed in table 2. After identifying those variables that were highly correlated, only a history of malignancy, the darbepoetin alfa to Hb ratio and the CACI were examined in a binary logistic regression analysis. A higher CACI and darbepoetin alfa to Hb ratio remained significantly associated with an MCV > 102 fl (table 2).

**Table 1:** Baseline characteristics

Variable	All (n = 150)	MCV ≤ 102 fl (n = 105)	MCV > 102 fl (n = 45)	P
Demographic				
Age (years)	65.0 ± 17.0	63.3 ± 17.6	68.9 ± 15.1	0.07
Time on dialysis (months)	34.5 ± 31.8	33.8 ± 34.4	36.4 ± 24.6	0.65
Male n (%)	76 (51)	58 (55)	18 (40)	0.09
Caucasian n (%)	135 (90)	91 (87)	44 (98)	0.04
Fistula as HD access n (%)*	45 (30)	33 (31)	12 (27)	0.56
Cause of ESRD n (%)				
Diabetes	51 (34)	40 (38)	11 (24)	0.11
Glomerulonephritis	29 (19)	18 (17)	11 (24)	0.30
Comorbidities n (%)				
Diabetes	63 (42)	45 (43)	18 (40)	0.75
Hypertension	114 (76)	79 (75)	35 (78)	0.74
Coronary artery disease	53 (35)	39 (37)	14 (31)	0.54
Peripheral vascular disease	33 (22)	23 (22)	10 (22)	0.97
Stroke	21 (14)	14 (13)	7 (16)	0.72
Cancer	25 (17)	11 (10)	14 (31)	< 0.01
Failed renal transplant	25 (17)	20 (19)	5 (11)	0.24
Charlson-Age Comorbidity Index	7 ± 3	6 ± 3	8 ± 3	0.01
Laboratory				
Mean MCV (fl)	99.1 ± 6.4	96.0 ± 4.6	106.3 ± 3.1	< 0.01
Albumin (g/L)	33.7 ± 3.5	34.0 ± 3.6	33.0 ± 3.3	0.14
Hemoglobin (Hb) (g/L)	108.6 ± 10.0	109.8 ± 10.0	105.7 ± 9.6	0.02
Hb btw. 100-120 g/L n (%)	103 (69)	73 (70)	30 (67)	0.65
Ferritin ug/L	729 ± 467	707 ± 454	782 ± 498	0.37
Tsat (%)	29.77 ± 11.80	28.05 ± 11.37	33.76 ± 11.93	< 0.01
Darbepoetin alfa to Hb ratio	5.37 ± 3.66	4.80 ± 3.49	6.69 ± 3.73	< 0.01
Medications				
Darbepoetin alfa dose mcg/week	43.0 ± 31.5	39.3 ± 31.1	51.6 ± 31.1	0.03
Receiving darbepoetin alfa n (%)^+^	141 (94)	97 (92)	44 (98)	0.27
Receiving intravenous iron n (%)^+^	137 (91)	96 (91)	41 (91)	0.95
Antiepileptic n (%)	5 (3)	1 (1)	4 (9)	0.01
On folate n (%)	148 (99)	103 (98)	45 (100)	0.81
On multivitamin	143 (95)	101 (96)	42 (93)	0.26

*Patients without a fistula were dialyzed using a tunnelled, cuffed catheter, except for one using an AV graft

+Received at least one dose during initial three months of study

Continuous variables are reported as means ± standard deviation

**Table 2:** Univariate and Multivariate Associations with MCV > 102 fl

	Univariate (OR [95% CI])	P	Multivariate (OR [95% CI])	P
Malignancy*	3.86 [1.59, 9.38]	0.003	2.12 [0.73, 6.17]	0.168
Darbepoetin alfa to Hb ratio	1.15 [1.04, 1.27]	0.005	1.19 [1.07, 1.32]	0.001
Charlson-Age Comorbidity Index	1.18 [1.04, 1.33]	0.012	1.23 [1.07, 1.40]	0.003
Transferrin saturation	1.04 [1.01, 1.07]	0.013	---	
Age 60 and above	3.34 [1.42, 7.86]	0.006	---	
Antiepileptic medication	10.15 [1.10, 93.51]	0.041	---	
Mean hemoglobin (g/L)	0.96 [0.93, 1.00]	0.025	---	
Weekly darbepoetin alfa dose (mcg)	1.01 [1.00, 1.02]	0.033	---	
Weekly weight adjusted darbepoetin alfa dose (mcg/kg)	3.21 [1.29, 7.98]	0.012	---	

* Hematologic and non-hematologic

Variables included in the multivariate binary regression analysis: Malignancy, Darbepoetin alfa to Hb ratio and the Charlson-Age Comorbidity Index.

### Mortality

**Figure 4:**
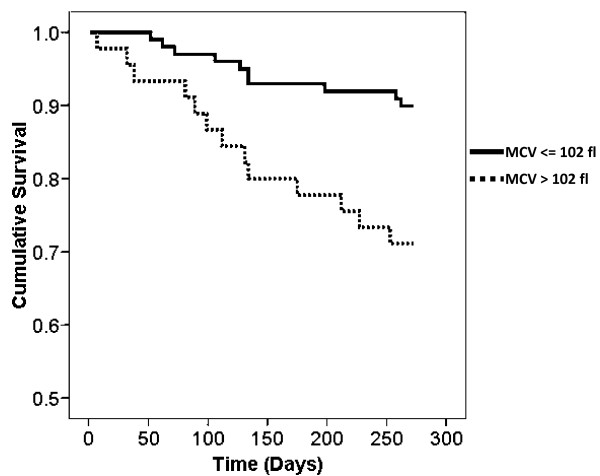
**Kaplan Meier survival for MCV > 102 fl**. Log rank p = 0.003

There were seven kidney transplants and 23 deaths prior to last follow up (September 1^st^, 2010). The mean MCV of all transplanted and deceased patients was 94.1 ± 4.4 fl and 102.1 ± 5.9 fl respectively. Causes of death are noted in table 3. At last follow up, an MCV > 102 fl was associated with increased mortality (log rank P = 0.003). Kaplan-Meier survival curves above and below an MCV of 102 fl are noted in figure 4. In a Cox survival analysis, an MCV > 102 fl was strongly associated with death (unadjusted HR 3.24 95% CI 1.42-7.39, P = 0.005). Other variables significantly associated with mortality in this study included age, stroke, history of malignancy at baseline, a higher CACI, lower mean albumin, higher mean ferritin, lower levels of uric acid and lower body weight. The darbepoetin alfa to Hb ratio was not associated with mortality in this study. After adjusting for the CACI, an MCV > 102 fl remained significantly associated with mortality (HR 2.47, 95% CI 1.07-5.71, p = 0.035).

**Table 3:** Cause of Death at Last Follow Up

	All (n = 150)	MCV < 102 fl (n = 105)	MCV > 102 fl (n = 45)
All Cause	23	10	13
Cardiac	7	3	4
Withdrawal	8	5	3
Sepsis	5	1	4
Cancer	2	1	1
Stroke	1	0	1

## Discussion

In this study of stable, chronic hemodialysis patients, we identified a high prevalence of unexplained macrocytosis associated with a higher CACI and higher ratios of weekly weight adjusted darbepoetin alfa to Hb. Macrocytosis both as a continuous variable and using a cutoff point of 102 fl was associated with higher all-cause mortality.

While the etiology of macrocytosis or its association with mortality in our population is unclear, several possibilities need to be considered. Macrocytosis was associated with a higher CACI at baseline that in turn, was associated with increased mortality. Perhaps macrocytosis is a response to underlying illness, and serves as a "window" into the bone marrow of an elderly dialysis patient with multiple comorbidities. The macrocytic response may not be a spontaneous development, rather, an effect of exogenous ESA administration to a chronically unwell patient. This would explain the association between MCV > 102 fl and higher darbepoetin alfa to Hb ratios, the latter reflecting ESA resistance perhaps due to underlying medical illness.

Regarding other possible causes, we did identify a univariate association between tsat and macrocytosis. Intravenous iron has been linked to increased mortality in dialysis patients [12,28,29], and has been suggested as a cause of macrocytosis [15,17]. However, in this study, mean iron dose over three months at baseline was not associated with an MCV > 102 fl. As well, there did appear to be a univariate association between macrocytosis and a history of malignancy, which in turn, was associated with mortality in this study. Macrocytosis occurs in certain malignancies, either independently or as a consequence of chemotherapy [30]. However, 11/14 macrocytic patients in our study with a history of malignancy did not have active cancer at the time of study, and no patients were actively receiving chemotherapy. Furthermore, only one death was directly attributable to cancer amongst the patients with an MCV > 102 fl.

A final consideration is that macrocytosis may be a manifestation of altered hematopoiesis. In one study of non-dialysis elderly patients, 25/49 with unexplained macrocytosis had dysplastic changes or findings consistent with MDS on bone marrow biopsy [31]. Alternatively, altered hematopoiesis may be due to high doses of ESAs. Macrocytosis due to suspected abnormal rapid cellular maturation in states of massive erythropoietic stimulation has been observed in rat models [22], and we did observe an association between darbepoetin alfa dose and MCV > 102 fl. However, altered hematopoiesis was not confirmed with bone marrow biopsy in this study. Furthermore, bone marrow dysplasia was not identified in an earlier study of dialysis patients treated with recombinant erythropoietin for 24 months who subsequently underwent bone marrow biopsy [32]. In addition, macrocytosis in dialysis patients has been identified prior to the introduction of ESAs [17].

As a predictor of increased mortality, macrocytosis does have advantages over other laboratory markers commonly used in dialysis patients. In our study, macrocytosis was a strong predictor of mortality evidenced by a high hazard ratio. In addition, as it is included in the routine CBC, measurement of the MCV requires no additional laboratory tests and is cheaper compared to conventional laboratory markers of mortality.

There are limitations to this study. With only 23 outcomes, we were unable to do extensive multivariate analyses without risk of over-fitting. Therefore, there is the possibility of residual confounding. However, this is primarily a pilot study and the main purpose is to stimulate hypothesis generation for future analyses. Another limitation is that the lack of serum B12 and red cell folate levels in all patients makes it difficult to completely exclude vitamin deficiency as a cause of macrocytosis. In addition, only a few patients had reticulocyte levels measured at baseline (a known cause of macrocytosis). However, 93 and 100% of patients with an MCV > 102 fl were on multivitamin and folate supplementation, and at six months, RBC folate and serum B12 levels were normal in over 90% of macrocytic patients (data not shown). In addition, a reanalysis at six months revealed comparable reticulocyte counts between macrocytic and normocytic patients, and an absolute reticulocytosis in only a few macrocytic patients (data not shown). Finally, as this is a single-centre study, we acknowledge that the findings of this study cannot necessarily be generalized to other dialysis populations. A recent study in a larger cohort of dialysis patients identified a lower mean MCV than our cohort, and did not find an association between MCV and mortality [23]. However, our study is unique as we analyzed MCV both as a continuous and categorical variable. Furthermore, our population is not unlike other dialysis cohorts with respect to its identified predictors of mortality. These predictors include age [33-36], stroke [33,34], cancer [33,34], albumin [35] and ferritin [37,38]. Nonetheless, our population may be unique with respect to a high prevalence of macrocytosis and validation with another dialysis population may increase the strength of our findings. In addition, if macrocytosis is associated with higher comorbidity it would fluctuate based on patient clinical status. Thus, a study using marginal structural models that adjust for time dependent confounding would be clinically relevant.

## Conclusions

Unexplained macrocytosis may be associated with mortality in stable, chronic hemodialysis patients. Future studies will need to be conducted in larger numbers of incident hemodialysis patients to determine the significance of this finding.

## Competing interests

The authors declare that they have no competing interests.

## Authors' contributions

KT and BK designed the study and collected and analyzed the data. KT drafted the initial article; BK, SS and KW critically revised the initial draft and made substantial contributions to the content of the article. All authors read and approved the final manuscript.

## Pre-publication history

The pre-publication history for this paper can be accessed here:

http://www.biomedcentral.com/1471-2369/12/19/prepub

## References

[B1] Eschbach JW Cook JD Scribner BH Finch CA Iron balance in hemodialysis patients Ann Intern Med 1977 87 710 713 931207 10.7326/0003-4819-87-6-710

[B2] Eschback JW Adamson JW Recombinant human erythropoietin: Implications for nephrology Am J Kidney Dis 1988 11 203 209 3278599 10.1016/s0272-6386(88)80150-1

[B3] Shaw AB Hemolysis in chronic renal failure BMJ 1967 2 213 215 10.1136/bmj.2.5546.213 6023106 PMC1841174

[B4] Hampers CL Streiff R Nathan DG Snyder D Merrill JP Megaloblastic hematopoiesis in uremia and in patients on long-term hemodialysis N Engl J Med 1980 276 226 233 10.1056/NEJM196703092761005 6019764

[B5] Eschbach JW Egrie JC Downing MR Browne JK Adamson JW Correction of the anemia of end-stage renal disease with recombinant human erythropoietin. Results of a combined phase I and II clinical trial N Engl J Med 1987 316 73 78 10.1056/NEJM198701083160203 3537801

[B6] Macdougall IC Novel erythropoiesis stimulating protein Semin nephrol 2000 20 375 381 10928340

[B7] Madore F Lowrie EG Brugnara C Lew NL Lazarus JM Bridges K Owen WF Anemia in hemodialysis patients: variables affecting this outcome predictor J Am Soc Nephrol 1997 8 1921 1929 9402095 10.1681/ASN.V8121921

[B8] Foley RN Parfrey PS Harnett JD Kent GM Murray DC Barre PE The impact of anemia on cardiomyopathy, morbidity and mortality in end-stage renal disease Am J Kidney Dis 1996 28 53 61 10.1016/S0272-6386(96)90130-4 8712222

[B9] Regidor DL Kopple JD Kovesdy CP Kilpatrick RD McAllister CJ Aronovitz J Greenland S Kalantar-Zadeh K Associations between changes in hemoglobin and administered erythropoiesis-stimulating agent and survival in hemodialysis patients J Am Soc Nephrol 2006 17 1181 1191 10.1681/ASN.2005090997 16565261

[B10] Lau JH Gangji AS Rabbat CG Brimble KS Impact of haemoglobin and erythropoietin dose changes on mortality: a secondary analysis of results from a randomized anaemia management trial Nephrol Dial Transplant 2010 25 4002 4009 10.1093/ndt/gfq330 20530806

[B11] Kaysen GA Muller HG Ding J Chertow GM Challenging the validity of the EPO index Am J Kidney Dis 2006 47 166.e1 166.e13 10.1053/j.ajkd.2005.09.013 16377397

[B12] Fishbane S Besarab A Mechanism of increased mortality risk with erythropoietin treatment to higher haematocrit targets Clin J Am Soc Nephrol 2007 2 1274 1282 10.2215/CJN.02380607 17942772

[B13] Zachee P Vermylen J Boogaerts MA Hematologic aspects of end-stage renal failure Ann Hematol 1994 69 33 40 10.1007/BF01757345 8061105

[B14] Afshar R Sanavi S Salimi J Ahmadzadeh M Hematological profile of chronic kidney disease (CKD) patients in Iran, in pre-dialysis stages and after initiation of hemodialysis Saudi J Kidney Dis Transpl 2010 21 368 371 20228535

[B15] Pollak VE Lorch JA Means RT Jr Unanticipated favorable effects of correcting iron deficiency in chronic hemodialysis patients J Investig Med 2001 49 173 183 10.2310/6650.2001.34044 11288758

[B16] Suega K Bakta M Dharmayudha TG Lukman JS Suwitra K Profile of anemia in chronic renal failure patients: comparison between predialyzed and dialyzed patients at the Division of Nephrology, Department of Internal Medicine, Sanglah Hospital, Denpasar, Bali, Indonesia Acta Med Indones 2005 37 190 194 16354939

[B17] Gokal R Weatherall DJ Bunch C Iron induced increase in red cell size in haemodialysis patients Q J Med 1979 48 393 401 542585

[B18] Fleming SJ Wilkinson JS Aldridge C Greenwood RN Muggleston SD Baker LR Cattell WR Dialysis-induced change in erythrocyte volume: effect on change in blood volume calculated from packed cell volume Clin Nephrol 1988 29 63 68 3359696

[B19] Bartels PC Helleman PW Soons JB Investigations on red cell size distribution histograms in subjects treated by maintenance haemodialysis J Clin Chem Clin Biochem 1990 28 113 118 2329314 10.1515/cclm.1990.28.2.113

[B20] Hofbrand V Provan D ABC of clinical haematology. Macrocytic anaemias BMJ 1997 314 430 433 9040391 10.1136/bmj.314.7078.430 PMC2125890

[B21] Paganini EP Latham D Abdulhadi M Practical considerations of recombinant human erythropoietin therapy Am J Kidney Dis 1989 14 19 25 2667348

[B22] Brecher G Stohlman F Jr Jacobson LO, Doyle M The macrocytic response to erythropoietin stimulation Erythropoiesis 1962 2 New York and London: Grune and Stratton 216 221

[B23] Pollak VE Lorch JA Shukla R Satwah S The importance of iron in long-term survival of maintenance hemodialysis patients treated with epoetin-alfa and intravenous iron: analysis of 9.5 years of prospectively collected data BMC Nephrol 2009 10 6 18 10.1186/1471-2369-10-6 19245700 PMC2671502

[B24] Charlson ME Pompei P Ales AL MacKenzie CR A new method of classifying prognostic comorbidity in longitudinal studies: development and validation J Chron Dis 1987 40 373 383 10.1016/0021-9681(87)90171-8 3558716

[B25] Beddhu S Bruns FJ Saul M Seddon P Zeidel ML A simple comorbidity scale predicts clinical outcomes and costs in dialysis patients Am J Med 2000 3 253 260 10.1016/s0002-9343(00)00371-5 10856407

[B26] Fernandez T Domack LB Montes D Pineiro R Landrum E Vital E Performance Evaluation of the Coulter LH 750 Hematology Analyzer Lab Hematol 2001 7 217 228

[B27] Final Report: The Canadian Enhancement of ICD-10 (International Statistical Classification of Diseases and Related Health Problems, Tenth Revision) http://www.cihi.ca 3376487

[B28] Besarab A Bolton WK Browne JK Egrie JC Nissenson AR Okamoto DM Schwab SJ Goodkin DA The effects of normal as compared with low hematocrit values in patients with cardiac disease who are receiving hemodialysis and epoetin N Engl J Med 1998 339 584 590 10.1056/NEJM199808273390903 9718377

[B29] Goodkin DA The normal hematocrit cardiac trial revisited Semin Dial 2009 22 495 502 10.1111/j.1525-139X.2009.00620.x 19650856

[B30] Thong KL Hanley SA McBride JA Clinical significance of a high mean corpuscular volume in nonanemic patients CMAJ 1977 117 908 910 PMC1880149 912617

[B31] Mahmoud MY Lugon M Anderson CC Unexplained macrocytosis in elderly patients Age Ageing 1996 25 310 312 10.1093/ageing/25.4.310 8831877

[B32] Sikole A Stojanovic A Polenakovic M Petrusevska G Sadikario S Saso R Jovanovski M How Erythropoietin Affects Bone Marrow of Uremic Patients Am J Nephrol 1997 17 128 136 10.1159/000169086 9096443

[B33] Goodkin DA Bragg-Gresham JL Koenig KG Wolfe RA Akiba T Andreucci VE Saito A Rayner HC Kurokawa K Port FK Held PJ Young EW Association of comorbid conditions and mortality in hemodialysis patients in Europe, Japan, and the United States: the Dialysis Outcomes and Practice Patterns Study (DOPPS) J Am Soc Nephrol 2003 14 3270 3277 10.1097/01.ASN.0000100127.54107.57 14638926

[B34] Miskulin D Bragg-Gresham J Gillespie BW Tentori F Pisoni RL Tighiouart H Levey AS Port FK Key comorbid conditions that are predictive of survival among hemodialysis patients Clin J Am Soc Nephrol 2009 4 1818 1826 10.2215/CJN.00640109 19808231 PMC2774950

[B35] Owen WF Jr Lew NL Liu Y Lowrie EG Lazarus JM The urea reduction ratio and serum albumin concentration as predictors of mortality in patients undergoing hemodialysis N Engl J Med 1993 329 1001 1006 10.1056/NEJM199309303291404 8366899

[B36] Qureshi AR Alvestrand A Divino-Filho JC Gutierrez A Heimburger O Lindholm B Bergstrom J Inflammation, malnutrition, and cardiac disease as predictors of mortality in hemodialysis patients J Am Soc Nephrol 2002 13 Suppl 1 128 36 11792759

[B37] Hasuike Y Nonoguchi H Tokuyama M Ohue M Nagai T Yahiro M Nanami M Otaki Y Nakanishi T Serum ferritin predicts prognosis in hemodialysis patients: the Nishinomiya study Clin Exp Nephrol 2010 14 349 355 10.1007/s10157-010-0288-x 20467772

[B38] Kalantar-Zadeh K Don BR Rodriguez RA Humphreys MH Serum ferritin is a marker of morbidity and mortality in hemodialysis patients Am J Kidney Dis 2001 37 564 572 10.1016/S0272-6386(01)80014-7 11228181

